# Flicker fusion thresholds as a clinical identifier of a magnocellular-deficit dyslexic subgroup

**DOI:** 10.1038/s41598-020-78552-3

**Published:** 2020-12-10

**Authors:** Jessica L. Peters, Edith L. Bavin, Alyse Brown, David P. Crewther, Sheila G. Crewther

**Affiliations:** 1 Department of Psychology and Counselling, La Trobe University, Melbourne, VIC 3086 Australia; 2 Intergenerational Health, Murdoch Childrens Research Institute, Melbourne, Australia; 3 MRC Cognition and Brain Sciences Unit, University of Cambridge, Cambridge, UK; 4 Centre for Human Psychopharmacology, Swinburne University of Technology, Melbourne, Australia

**Keywords:** Neuroscience, Psychology

## Abstract

The magnocellular-dorsal system is well isolated by high temporal frequency. However, temporal processing thresholds have seldom been explored in developmental dyslexia nor its subtypes. Hence, performances on two, four-alternative forced-choice achromatic flicker fusion threshold tasks modulated at low (5%) and high (75%) temporal contrast were compared in dyslexic and neurotypical children individually matched for age and intelligence (8–12 years, *n* = 54 per group). As expected, the higher modulation resulted in higher flicker fusion thresholds in both groups. Compared to neurotypicals, the dyslexic group displayed significantly lower ability to detect flicker at high temporal frequencies, both at low and high temporal contrast. Yet, discriminant analysis did not adequately distinguish the dyslexics from neurotypicals, on the basis of flicker thresholds alone. Rather, two distinct dyslexic subgroups were identified by cluster analysis – one characterised by significantly lower temporal frequency thresholds than neurotypicals (referred to as ‘Magnocellular-Deficit’ dyslexics; 53.7%), while the other group (‘Magnocellular-Typical’ dyslexics; 46.3%) had comparable thresholds to neurotypicals. The two dyslexic subgroups were not differentially associated with phonological or naming speed subtypes and showed comparable mean reading rate impairments. However, correlations between low modulation flicker fusion threshold and reading rate for the two subgroups were significantly different (*p* = .0009). Flicker fusion threshold performances also showed strong classification accuracy (79.3%) in dissociating the Magnocellular-Deficit dyslexics and neurotypicals. We propose that temporal visual processing impairments characterize a previously unidentified subgroup of dyslexia and suggest that measurement of flicker fusion thresholds could be used clinically to assist early diagnosis and appropriate treatment recommendations for dyslexia.

## Introduction

Developmental Dyslexia is a heterogenous neurodevelopmental disorder affecting ~ 10% of individuals, who are characterized by impaired reading accuracy, speed and comprehension, i.e. dysfluency^1^. While dyslexia has most often been associated with impairments in phonological processing^2,3^, the three most common models of dyslexia^4–6^ propose several distinct subtypes. (1) A phonological deficit subtype also referred to as dysphonesia; (2) visually-based subtypes characterized by either an orthographic (i.e., dyseidesia or surface dyslexia^4,5^) or rapid naming speed deficit^6^; (3) combination subtypes with both phonological and orthographic deficits (referred to as dysphoneidesia or mixed dyslexia^4,5^) or both phonological and rapid naming deficits (referred to as a ‘double-deficit’^6^); and (4) a ‘no-deficit’ subtype where those with dyslexia do not display phonological, orthographic or naming speed deficits despite significant reading difficulties.

Although less accepted, converging lines of evidence also implicate visual impairments in dyslexia across mechanisms specifically associated with the magnocellular (M) pathway of the retinocortical dorsal visual stream^7–16^, with some suggesting that M-based impairments may only be experienced by a subgroup of dyslexic individuals^17^. However, the Magnocellular Theory of Dyslexia has remained somewhat contentious due to variability in findings and difficulty isolating the faster conducting M pathway that contributes to both the dorsal and ventral visual streams^18–23^. As yet, there is no well-established psychophysical measure of both spatial and temporal aspects of magnocellular-dorsal stream function that could be employed clinically to aid diagnosis of dyslexia and help guide appropriate interventions.

Indeed, temporal threshold manipulations, as compared to other proxies for M function properties such as low spatial frequency or low contrast performance have seldom been used to explore dyslexia. This is despite primate single cell physiological studies showing that the M stream is best isolated by stimuli presented at fast temporal frequencies^24–26^, and evidence from Klistorner and colleagues^27^ using multifocal Visually Evoked Potentials to show distinct temporally limiting stimulus recovery characteristics of the M and Parvocellular (P) pathway contributions to the primary visual cortex (V1) in humans (see also^28,29^). Where moving stimuli, such as in motion coherence tasks, have been used to study dyslexia, paradigms have usually been studied at frequencies well below the ~ 15 Hz needed to isolate M from P contributions in primates^26^ and humans^11,30–32^. Moreover, the low contrast and spatial deficits often seen in dyslexic individuals have been reported to become more apparent at increasingly higher temporal frequencies^33,34^, though only under certain conditions^35^. Hence, we propose that it may be the temporal processing properties of the magnocellular-dorsal system that is of greatest importance to reading, and that psychophysical tasks of temporal processing thresholds may prove to be the most valid and opportunistic, non-invasive tests of magnocellular sensitivity currently available.

The simplest test of temporal processing threshold for neural recovery to repeated stimulation is the Flicker Fusion Threshold (FFT) task which assesses the absolute temporal processing threshold at which rapid modulation of flickering light is no longer detectable– i.e., the point of fusion^36,37^. The high temporal and extremely low spatial properties of an achromatic FFT task means that the point of fusion is set by the speed of neural recovery of the faster M cells in the primary visual cortex^38,39^. Indeed, Brown and colleagues^28^ demonstrated in neurotypical adults that temporal processing of achromatic low and high contrast FFTs correlate with M (but not P) nonlinear visual evoked potentials. The point of achromatic fusion is reported to occur between 35–64 Hz and is contingent on the luminance, size of lighting source and depth of modulation^36,37,40^, and age^41,42^. FFTs have also been shown to be related to auditory temporal resolution and word decoding ability in typical readers^43,44^, but to our knowledge, only five studies have compared the FFT performance, also referred to as critical flicker fusion, of individuals with dyslexia and typical readers^45–49^. Four of these studies found that on flicker fusion tasks using M-preferred stimuli, dyslexics displayed significantly lower temporal frequency thresholds (i.e., lower sensitivity) as compared to a typical reader group, but that FFTs for P-preferred tasks were not different where evaluated (see Supplementary Table S1 for a summary of each study).

Thus, the present study aimed to clarify the importance of rate of magnocellular processing for reading performance using FFTs, and to establish the utility of FFT tasks as a potential clinical measure of dyslexia, either in general or as a classifier of subtypes. Hence, we have compared the achromatic temporal processing thresholds of dyslexic children and neurotypical children (with normal reading skills), individually matched on age and nonverbal intelligence. The two achromatic FFT tasks modulated at high (75%) and low (5%) contrast were adopted from Brown and colleagues^28^ and used as measures of the temporal frequency threshold (i.e., Hz) of M processing efficiency. Classification of dyslexic subtypes based on the presence and or absence of phonological awareness and rapid naming speed deficits (referred to as the Double-Deficit Hypothesis), was employed to identify if impairments in temporal processing of the visual system may be related to these previously proposed subtypes.

Specifically, the study aimed to explore the following research questions:

(i) Can FFT performance using low and high contrast achromatic FFT tasks dissociate groups of dyslexic and neurotypical children? And are there subgroups of dyslexic children with and without temporal processing (i.e., FFT) deficits?
(ii) If subgroups are present, are they associated with previously described subtypes of dyslexia?
(iii) Can FFT be used to discriminate dyslexic children with temporal deficits from neurotypical children and hence be established as a clinically useful test of magnocellular-dorsal functioning?

## Results

### Data analysis

An a priori power analysis indicated that with a total of 90 participants (e.g., 45 per group) there was 95% power to detect a moderate effect size at *p* = .05. Data screening of the complete dataset identified several outliers that were just outside the normal distribution. These outliers were treated using the Winsorization method, i.e., they were recoded to the largest value within the normal distribution to reduce the influence on parametric statistical analyses^50^. Normality was confirmed via assessment of skewness and kurtosis values, Shapiro–Wilk values, and visual inspection of histograms, Normal Q-Q plots and box plots. Cook distances were used to identify influential outlying data points in the correlational analyses; these data points were then removed from the relevant correlations. Preliminary analyses revealed no further violations of assumptions for the conducted analyses. Bonferroni adjustments were applied to the alpha level where multiple comparisons were conducted.

### Can differences in temporal processing dissociate groups of dyslexic and neurotypical children?

Results of the multivariate analysis of variance show dyslexic children to have significantly lower flicker fusion sensitivity (i.e., slower temporal processing) at both 75% and 5% contrast as compared to the neurotypical children, both in the main multivariate analysis and subsequent univariate analyses, with moderate effect sizes (See Table 1).

**Table 1:** Comparison of flicker fusion thresholds in dyslexic and neurotypical children.

	Dyslexic children (*n* = 54)	Neurotypical children (*n* = 54)	*F*	*df*	*p*	*d*
	*M (SD)*	*M (SD)*				
Multivariate analysis	-	–	4.36	2, 105	.015*	0.50
75% FFT	48.18 (4.27)	50.41 (4.58)	6.93	1, 106	.010**	0.51
5% FFT	45.16 (4.52)	47.22 (3.62)	6.56	1, 106	.012*	0.50

Bonferroni adjusted alpha level of *p < .016, **p ≤ .01; Cohen’s *d* ≥ 0.2, *d* ≥ 0.5, and *d* ≥ 0.8, represent small, medium, and large effect sizes, respectively.

Discriminant function analysis was used to identify whether FFTs could dissociate dyslexic children from neurotypicals. Results demonstrated that FFT sensitivity significantly differentiated dyslexic and neurotypical children, λ = 0.923, χ^2^(2) = 8.367, *p* = 0.015, *R*^2^ = 0.077, with both the high contrast (*r* = 0.888) and low contrast tasks (*r* = 0.863) loading highly onto the discriminant function. However, the classification accuracy of the model was low. In total, 56.6% of dyslexic and 61.1% of neurotypical children were accurately classified. Overall, the model showed only a 58.9% classification accuracy. Thus, further analysis was conducted to identify if there were subgroups in the dyslexic sample.

### Are there subgroups of dyslexic children with and without temporal processing deficits?

**Figure 1:**
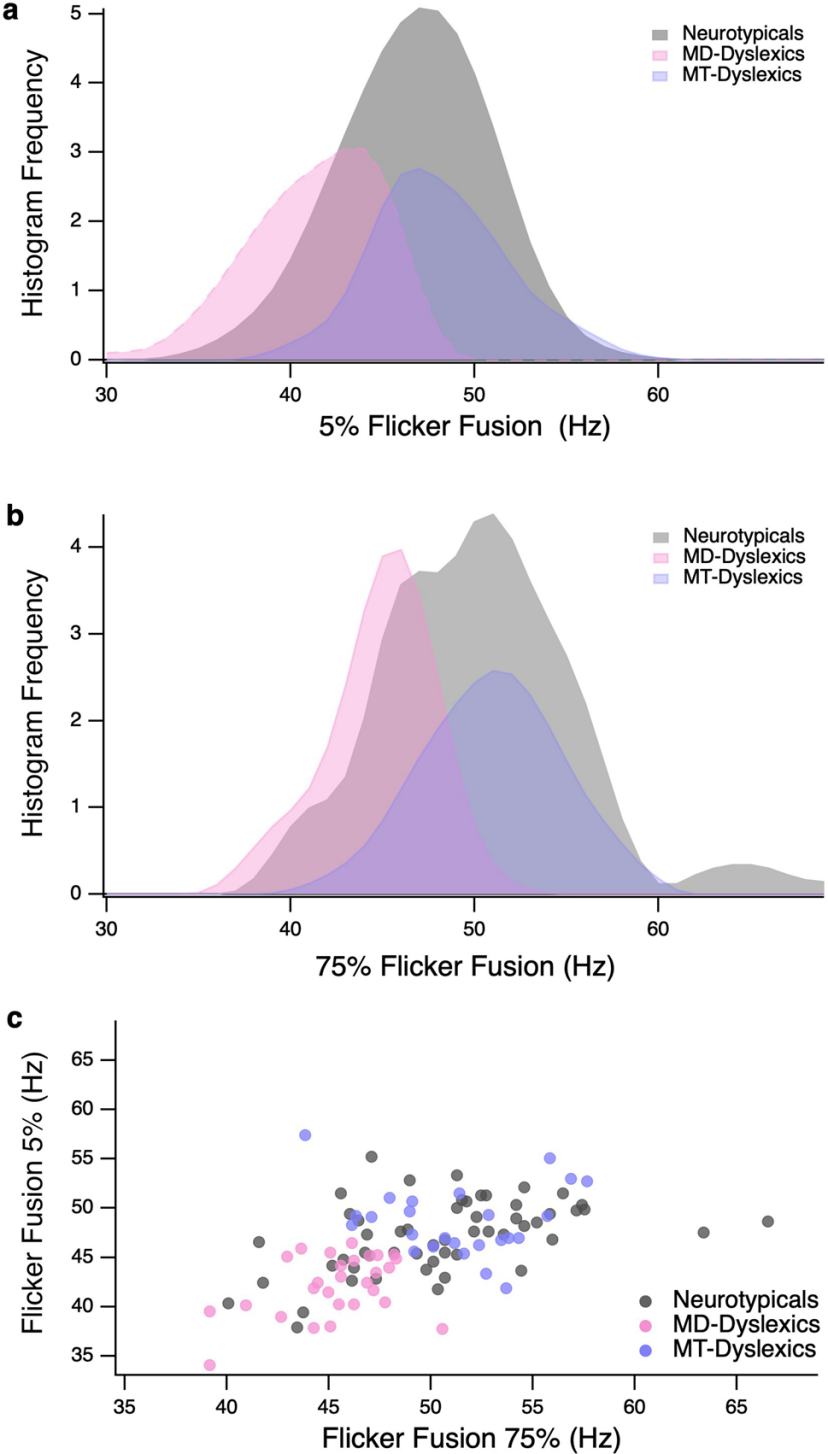
Flicker Fusion Threshold (FFT) distribution of the dyslexic subgroups identified via cluster analysis and of the Neurotypicals; Frequency distributions of Subgroup A (‘Magnocellular-Deficit Dyslexics’), Subgroup B (Magnocellular-Typical Dyslexics’) and Neurotypicals for (**a**) the Low Contrast (5%) FFT Task; and (**b**) the High Contrast (75%) FFT Task. (**c**) Scatterplot of Low and High Contrast FFT performance of the identified dyslexic clusters and neurotypicals. Note. Neurotypicals have been included in (**a**) and (**b**) for comparative purposes and were not included in the cluster analysis.

Two-step cluster analysis based on the high and low contrast FFT performance of the dyslexic sample revealed a two-cluster solution. The analysis used a log-likelihood distance measure approach with Schwarz’s Bayesian Criterion^51^ and number of clusters were not specified in advance. The average silhouette measure = 0.60 (i.e., a measure of cluster cohesion and separation) indicated good cluster quality for the two clusters, as shown in Fig. 1 and Table 2. Subgroup A (*n* = 29; 53.7%) demonstrated FFT impairments and so were termed Magnocellular-Deficit Dyslexics (MD-Dyslexics), while Subgroup B (*n* = 25; 46.3%), who demonstrated unimpaired FFTs, were labelled Magnocellular-Typical Dyslexics (MT-Dyslexics). Overall, the low contrast (5% modulation) task was found to be the most discriminative predictor of cluster membership.

**Table 2:** Comparison of neurotypical and dyslexic subgroups for flicker fusion, age, nonverbal intelligence, and reading measures.

	a. MD-Dyslexics *n* = 29		b. MT-Dyslexics *n* = 25		c. Neurotypical children *n* = 54		*F* (2, 105)	*p*	*d*	Tukey HSD Post hoc
	*M (SD)*	Range	*M (SD)*	range	*M (SD)*	range				
75% FFT	45.38 (2.66)	39.17–50.57	51.29 (3.54)	43.85–57.68	50.42 (4.57)	40.09–59.68	19.46	< .001	1.22	a < b**, a < c**, b = c
5% FFT	41.94 (3.23)	34.12–46.45	48.56 (3.31)	41.88–55.09	47.20 (3.64)	37.93–55.21	30.04	< .001	1.51	a < c**, a < c**, b = c
Age	9.96 (1.20)	8.00–12.25	10.34 (1.20)	8.08–12.92	9.86 (1.13)	8.00–12.17	1.46	.237	0.33	–
Nonverbal Intelligence	103.66 (8.87)	88–121	105.48 (6.96)	89–118	106.70 (10.19)	85–121	1.04	.357	0.28	–
Reading Accuracy	77.32 (9.15)	69–99	76.80 (6.27)	69–90	100.74 (10.25)	80–120	88.78	< .001	2.61	a < c**, b < c**, a = b
Reading Rate	74.56 (7.44)	69–90	76.44 (7.29)	69–90	99.96 (9.89)	80–124	103.96	< .001	2.84	a < c**, b < c**, a = b
Reading Comprehension	87.96 (14.45)	69–117	94.04 (12.50)	69–113	104.72 (11.80)	81–131	17.53	< .001	1.16	a < c**, b < c*, a = b
Phonological Awareness	87.81 (12.67)	70–116	87.20 (10.61)	70–110	103.33 (11.16)	85–125	25.34	< .001	1.40	a < c**, b < c**, a = b
Rapid Naming	82.78 (12.03)	61–104	84.00 (9.31)	69–104	95.26 (12.41)	69–118	13.59	< .001	1.04	a < c**, b < c**, a = b

To account for the multiple comparisons, a Bonferroni adjustment to the alpha level (*p* = .006) was applied; Cohen’s *d* ≥ 0.2, *d* ≥ 0.5, and *d* ≥ 0.8, represent small, medium, and large effect sizes, respectively; FFT scores are reported in hertz; all neuropsychological measures are reported as Standard Scores. For post-hoc analyses *p < .05, ** *p* < .001.

Results of the subsequent ANOVAs show the two dyslexic subgroups did not differ in age or nonverbal intelligence, nor text reading, phonological awareness nor rapid naming measures, confirming that the identified clusters were not an artifact of known factors (See Table 2). Rather, MD-Dyslexics were specifically characterized by significantly lower temporal thresholds (i.e., slower temporal processing) across FFT for both contrast modulation tasks compared with MT-Dyslexics and neurotypical children. In contrast, MT-Dyslexics demonstrated temporal thresholds that were equivalent to the neurotypical children at both contrast modulations, though their reading, rapid naming scores and phonological awareness were significantly reduced.

### Are temporal processing deficits associated with previously described subtypes of dyslexia?

The total dyslexic sample (*N* = 54) was classified into four subtypes based on performances at least 1 SD below age expectations on either the rapid naming composite (Naming Speed Deficit; *n* = 12), elision task (Phonological Deficit; *n* = 10), both tasks (Double-Deficit; *n* = 18), or neither task (No-Deficit; *n* = 14) according to criteria provided by Wolf and Bowers^6^. This permitted investigation to determine if these subtype classifications could predict those dyslexic participants identified with and without temporal processing impairments (i.e., FFT deficit) in the cluster analysis. Subtype classification was then confirmed via ANOVA (see Supplementary Information), and the presence (or absence) of a temporal processing deficit, based on the results of the cluster analysis, was entered as the dependent variable. The results of a direct logistic regression were not significant, χ^2^ (3, *N* = 54) = 4.88, *p* = 0.181, indicating that temporal processing impairments per se are not specifically associated with the subtypes proposed by the Double-Deficit Hypothesis (See also Supplementary Table S2 and S3).

### How do temporal visual processing thresholds relate to reading skills?

As shown in Table 3, one-tailed Pearson correlational analyses revealed that better performance on the high contrast FFT task correlated significantly with better low contrast FFT, nonverbal intelligence, reading accuracy, reading rate, phonological awareness, and rapid naming performances in neurotypical children.

**Table 3:** Correlations between reading skills and flicker fusion thresholds for each group.

	a. MD-Dyslexics *n* = 29		b. MT-Dyslexics *n* = 25		c. Neurotypicals *n* = 54	
	5% FFT (*M* = 41.94)	75% FFT (*M* = 45.38)	5% FFT (*M* = 48.56)	75% FFT (*M* = 51.29)	5% FFT (*M* = 47.20)	75% FFT (*M* = 50.42)
5% FFT	–	.415*	–	.177	–	.498**
Nonverbal Intelligence	− .025	.161	.477**	.26	.162	.287*
Reading Accuracy	.182	.365*	.025	− .111	.122	.435**
Reading Rate	.343*	.337*	− .542**	.083	.053	.349**
Reading Comprehension	.278	− .158	.244	− .230	.092	.082
Phonological Awareness	.326*	.616**	.364*	.422*	.172	.429**
Rapid Naming	.313	.334*	− .313	− .304	.127	.284*

*p < .05, ** *p* < .01; According to Cohen’s guidelines, *r* ≥ 0.10, *r* ≥ 0.30, and *r* ≥ 0.50, represent small, medium, and large effect sizes, respectively^68^; standard scores are used for all clinical tasks. FFTs are reported in Hz.

MD-Dyslexics showed a similar pattern of correlations, with high contrast FFT also correlating positively with low contrast FFT, reading accuracy, rate, phonological awareness and rapid naming performances, and low contrast FFT positively correlating with reading rate and phonological awareness. In the MT-Dyslexic subgroup, high and low contrast FFTs also positively correlated with phonological awareness, while low contrast FFT also correlated positively with nonverbal intelligence. Moreover, MT-Dyslexics, in contrast to MD-Dyslexics, showed a negative correlation between 5% FFT and reading rate. The difference in correlations between MT and MD subgroups, tested by applying Fisher’s transformation between the groups for FFT 5% and reading rate, showed a significant difference (Z = 3.33, *p* = 0.0009, two-tailed).

**Figure 2:**
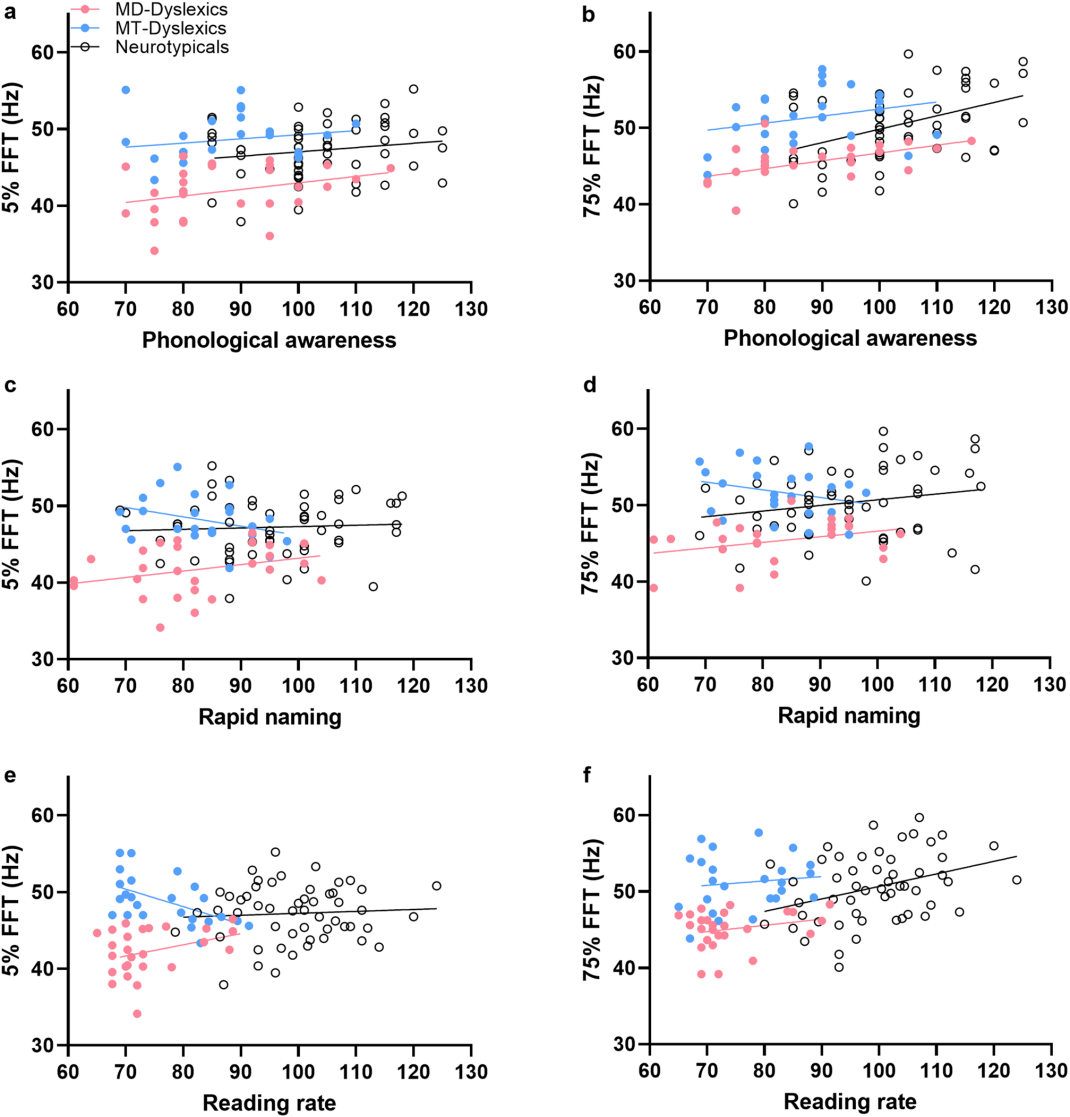
Correlations between Flicker Fusion Thresholds (FFTs) and reading measures; Low Contrast (5%) FFTs and (**a**) Phonological awareness; (**c**) Rapid Naming; (**d**) Reading Rate; and High Contrast (75%) FFTs with (**b**) Phonological awareness; (**d**) Rapid naming; (**f**) Reading rate.

MT-Dyslexics also showed a negative trend in the relationship between FFTs and rapid naming. Scatterplots for phonological awareness, rapid naming, and reading rate are shown in Fig. 2. They indicate that the correlational differences reported between the two dyslexic subgroups are not due to difference in range of performances on phonological awareness, rapid naming and reading rate.

### Is FFT a valid clinical identifier of dyslexic children with temporal deficits?

Results of a second discriminant analysis showed that FFTs significantly differentiated the MD-Dyslexic subgroup from the neurotypical children, λ = 0.631, χ^2^(2) = 36.411, *p* < 0.001, canonical *R*^2^ = 0.370, with both the high contrast (*r* = 0.786) and low contrast (*r* = 0.916) loading highly onto the discriminate function. Overall, the model showed strong classification accuracy (79.3%), with 82.14% (sensitivity) of MD-Dyslexics and 77.78% (specificity) of neurotypicals accurately classified.

## Discussion

Thresholds of temporal processing have rarely been used to explore the Magnocellular Deficit Theory of Dyslexia, despite strong evidence indicating that the M stream is best isolated by its fast firing recovery rates, rather than just contrast and spatial properties^24–29^. Thus, the current study investigated temporal processing thresholds of dyslexic and typical children using FFTs, at both low and high contrast, to comprehensively establish a magnocellular-temporo-spatial test that could be utilized clinically.

Our findings extend on those from previous FFT studies of dyslexia^45–48^ by showing significant heterogeneity in dyslexic FFT performances and, more importantly, establishing the presence of two distinct subgroups – one characterized by impaired magnocellular-temporal processing thresholds (MD-Dyslexics) and the other by threshold levels equivalent to those of neurotypicals (MT-Dyslexics). The finding that 53.7% of the dyslexic sample were classified as MD-Dyslexics is comparable with the prevalence reported for phonological (47–62%) and rapid naming (19–44%) subtypes of dyslexia^52^. Although no previous FFT study has analysed the presence of dyslexic subgroups with and without temporal deficits, research from our lab has shown that 43–50% of dyslexic children demonstrated FFTs 1 SD below matched controls^45^. Similarly, McLean, et al. ^48^ identified that 42.5% of their dyslexic children performed 1 SD below matched controls on M-based temporal thresholds. Together, the consistency of these findings suggests that almost half of dyslexic individuals are likely to be characterised by magnocellular-temporal processing impairments.

Our results indicate that the presence of temporal processing impairments are not necessarily related to the phonological, rapid naming, double-deficit, or no-deficit subtypes of dyslexia that are proposed by Wolf and Bowers’ Double-Deficit Hypothesis^6^ and so provide evidence for the identification of a new subgroup of dyslexia characterised by visual temporal processing differences. Our results compliment the findings of several studies showing little association between tests of M functioning and specific subtypes of dyslexia^53,54^, however, others studies have reported a link between M functioning and phonological and double-deficit subtypes^55–58^, indicating a clear need for further research. Inclusion of orthographic processing tasks, for example, would enable future FFT research to further explore dyseidetic and surface subtypes^4,5^.

The MD-Dyslexic and neurotypical groups showed similar correlation patterns. FFT performance, particularly high contrast FFT, was related to performance in almost all reading measures assessed (reading accuracy and rate, phonological awareness, rapid naming, but not reading comprehension). By comparison, the FFTs of MT-Dyslexics showed a different relationship pattern with reading skills: 5% contrast FFT was negatively related to reading rate (i.e., speed); there were also non-significant negative trends between FFTs and rapid naming, a task also reliant on speed. However, like the other two groups, FFTs of the MT-Dyslexics correlated with phonological awareness. This is consistent with several other papers that show a relationship between M functioning and phonological skills^44,46,59–62^. While it is not fully clear what other factors differentially characterise MD-Dyslexics and MT-Dyslexics, several possibilities can be speculated from the current findings. Reading rate and low contrast temporal processing were positively related in MD-Dyslexics, but negatively related in MT-Dyslexics. This dissociation was statistically significant and provides novel evidence indicative of a relationship between temporal processing speed and reading speed. For MT-Dyslexics, despite efficient temporal processing speed, performance on speed-based measures (i.e., reading rate and naming speed) are likely to be related to additional factors not measured in the current study, such as speed of articulation, automaticity in accessing the multiple cognitive processes required by both tasks, and other cognitive processes, including working memory.

In the current study, MD-Dyslexics demonstrated flicker frequency thresholds that were on average 10–13% (~ 5 to 6 Hz) lower than MT-Dyslexics and neurotypicals (as shown in Table 2). Reduced FFT performance can be used to discriminate MD-Dyslexics from neurotypical populations with good sensitivity (82.14%) and specificity (77.78%). Although the clinical significance of a specific M-based temporo-spatial psychophysical task has rarely been considered in past research, similar classification accuracy was reported for adult dyslexics by Talcott^46^, as shown in Supplementary Table S1. Our results provide initial evidence for the clinical applicability of FFT tasks as reliable measures of magnocellular-temporal efficiency that could be used to aid assessment and diagnosis of one subgroup of dyslexia. As interventions that target other properties of the M stream (i.e., motion and contrast) show strong efficacy in improving aspects of reading, and in particular reading rate^63,64^, future research should also consider evaluating the utility of FFT and other temporal processing training programs for dyslexia.

In summary, the findings of the current study establish that a subgroup of dyslexic children (MD-Dyslexics) are specifically characterized by a significant impairment in magnocellular-temporal processing thresholds. The presence of this temporal impairment was not better categorized by the presence or absence of phonological awareness and/or rapid naming impairments as is commonly used to classify dyslexic subtypes in past research. It was also not an artifact of differences in age, nonverbal intelligence, nor severity or pattern of reading impairments between the MD-Dyslexic and MT-Dyslexic subgroups as they were comparable on reading, phonological awareness and rapid naming performances. Rather, for MD-Dyslexics, poorer FFTs, particularly high contrast FFT, was related to worse reading accuracy, reading rate, phonological awareness and rapid naming outcomes. In contrast, the FFTs of MT-Dyslexics were much less related to reading outcomes. Thus, we propose that temporal processing impairments characterize a previously unidentified subtype of dyslexia. This subtype can be easily identified using FFT tasks with good clinical accuracy (79.3%). FFTs tasks should therefore be considered as a valid non-invasive test of magnocellular temporal efficiency for dyslexia, that could be clinically used to assist diagnosis and appropriate treatment recommendations. FFT tasks could also prove useful in identifying pre-reading children at risk of developing dyslexia, though further research is required.

## Methods

### Participants and procedure

A total of 58 dyslexic children and 70 neurotypical children with normal reading ability between the ages of 8–12 years, from Grades 3–6, participated in the study. Of those, 54 dyslexics and 54 neurotypicals were able to be one-to-one matched within ± 0.73 SD of nonverbal intelligence, and within ± 1 year of age, and these 108 children were included in the analyses (See Table 4 for descriptives and group comparisons).

**Table 4:** Descriptives and comparisons of dyslexic and neurotypical children.

	Dyslexic children (*N* = 54)			Neurotypical children (*N* = 54)			*t* (106)	*p*	*d*
	*M*	*SD*	*Range*	*M*	*SD*	Range			
Age	10.14	1.20	8.00–12.92	9.86	1.13	8.00–12.17	− 1.25	.219	0.24
Nonverbal intelligence	104.50	8.02	88–121	106.70	10.19	85–121	1.5	.215	− 0.24
Reading Accuracy	77.08	7.85	69–99	100.74	10.25	80–120	13.38	< .001**	− 2.59
Reading Rate	76.00	8.71	69–103	99.96	9.89	80–124	13.22	< .001**	− 2.57
Reading Comprehension	91.53	15.28	69–131	104.72	11.79	81–131	5.01	< .001**	− 0.97
Phonological Awareness	87.52	11.61	70–116	103.33	11.16	85–125	7.15	< .001**	− 1.39
Rapid Naming	83.14	11.14	58–104	95.26	12.41	69–118	5.24	< .001**	− 1.03

All scores, except for age, are reported as standard scores; Cohen’s *d* ≥ 0.2, *d* ≥ 0.5, and *d* ≥ 0.8, represent small, medium, and large effect sizes, respectively.

Participants were recruited and assessed at Melbourne metropolitan primary schools and an extra-curricular summer education program for those with reading difficulties between 2017 and 2018. All participants had normal intelligence (Standard score ≥ 85 for age on the Raven’s Coloured Progressive Matrices test), normal or corrected-to-normal vision and hearing, and English as their primary language. Children with known medical and neurodevelopmental disorders other than dyslexia (e.g., ASD, ADHD) were excluded. To be included in the dyslexic sample, participants required (1) a history of reading difficulties as reported by teachers or parents and/or a formal diagnosis of dyslexia, and (2) reading performance at least 1.25 SD^52^ below age-standardized norms in one or more area of reading (text reading accuracy, rate and/or comprehension) on the York Assessment of Reading for Comprehension—Primary Reading (YARC^65^), as confirmed by a psychologist on the research team. Parents of participants provided written informed consent for their child to engage in the study and all children who participated provided verbal assent. Testing occurred in a quiet, light-controlled room either at the child’s school or at the site of the educational program, with tasks administered in randomized order. The experiment was performed in accordance with relevant guidelines and regulations and with ethics approval granted by the La Trobe University Faculty Human Ethics Committee and the Victorian State Department of Education.

## Materials

### Neuropsychological tests

Nonverbal intelligence was assessed using the Raven’s Coloured Progressive Matrices^66^. The YARC was used to assess text reading accuracy, rate and comprehension skills^65^. The elision subtest, a measure of phonological awareness, and the rapid symbolic naming composite, consisting of letter and number rapid naming tasks, from the Comprehensive Test of Phonological Processing, 2nd Edition^67^ (CTOPP-2) were administered to investigate relationships between FFT and reading-related skills and to classify dyslexic participants into subtypes as based on the Double-Deficit Hypothesis^6^. All psychometric measures are reported as standardized scores obtained from the norm referenced instruments.

### Temporal processing thresholds

Two achromatic FFT tasks, modulated at high (75%) and low (5%) contrast in separate experimental tasks, were used. Four LEDs (A-Bright Industrial Co., China, part AL-513W3c-003 white) conveyed light into separate 6 mm diameter optic fibre light guides which were presented flush in a free-standing wooden panel in a diamond-array subtending 1.0◦, center-to-center, at the eye. The task was designed with VPixx software and flicker modulation was controlled via a DATAPixx device (10 kHz sampling allowed for smooth temporally modulated sinusoidal waveforms with frequencies in excess of 100 Hz). A gaussian temporal envelope (Full width at half maximum = 480 ms) was used to smooth the onset and offset of the flicker to prevent the alerting of change sensitive mechanisms and each light was calibrated for luminance. Each task consisted of a four-alternative forced-choice deign with 32 trials and used a Parameter Estimation by Sequential Testing. For further details about task design, see Brown^28^.

Participants completed the task at a viewing distance of 60 cm in a dimly lit room. They were instructed that one LED light per trial (demarcated by a high-pitched beep) would flicker for 3 s and at the end of the trial (indicated by a low-pitched beep) they were required to indicate which light source they saw flicker or guess when they were unsure. Prior to task commencement, participants were provided a practice session to familiarize them. During the tasks, the start of each trial was manually controlled by the experimenter to ensure participants were attending, and the onset of a trial began with a high pitch beep and finished with a low pitch. The order of high and low contrast conditions was counterbalanced to control for practice effects.

## Supplementary information

Supplementary information.

## Data Availability

The data that support the findings of this study are available from the corresponding author, upon reasonable request.

## Abbreviations

FFT: Flicker fusion threshold
M: Magnocellular
P: Parvocellular
MD-Dyslexic: Magnocellular-deficit dyslexic
MT-Dyslexic: Magnocellular-typical dyslexic
