# Supplementary material for: Flicker fusion thresholds as a clinical identifier of a magnocellular-deficit dyslexic subgroup
Source: Sci Rep. 2020 Dec 10;10:21638. doi: 10.1038/s41598-020-78552-3 (PMC7730401; doi:10.1038/s41598-020-78552-3)
Supplement: Supplementary file 1 — Supplementary information. [file 41598_2020_78552_MOESM1_ESM.docx]

Supplementary Information

Flicker Fusion Thresholds as a Clinical Identifier of a Magnocellular-Deficit Dyslexic Subgroup

**Peters, J.,^1*^ Bavin, E. L.,^1,2^ Brown, A.,^1,3^ Crewther, D. P.,^1,4^ & Crewther S. G.^1^**

^1^Department of Psychology and Counselling, La Trobe University, Melbourne, Australia;

^2^Intergenerational Health, Murdoch Childrens Research Institute, Melbourne, Australia;

^3^MRC Cognition and Brain Sciences Unit, University of Cambridge, United Kingdom;

^4^Centre for Human Psychopharmacology, Swinburne University of Technology, Melbourne, Australia.

Corresponding Author:

Jessica Peters

E: j.peters@latrobe.edu.au

| Supplementary Table S1.  *Studies Comparing Flicker Fusion Thresholds in Dyslexic and Typical Readers* | | | | |
| --- | --- | --- | --- | --- |
| Citation | Participants | | Task Description | Results |
|  | *N* | Age M (SD), Range |  |  |
| Chase and Jenner (1993) | 7 dyslexics;  8 typical readers | 17-22 | Visual temporal processing thresholds assessed using 4 trials of paired visual stimuli presented spatiotemporally to result in a ‘fused’ overlapping composite image at threshold. Participants indicated when the display no longer appeared to flicker.  Magnocellular conditions:  Shape, apparent movement, letters.  Parvocellular condition: equiluminant red/green colour. | Across groups, flicker fusion thresholds for magnocellular tasks were faster than parvocellular tasks (31.9 Hz versus 16.7 Hz, respectively).  Magnocellular condition:  Flicker Fusion thresholds were significantly lower in dyslexics (M = 26.1 Hz) as compared to controls (M = 38.4 Hz)  Parvocellular condition:  No differences between dyslexics and controls (~15.87 Hz versus ~17.24 Hz, respectively, as estimated from figure) |
| Talcott et al. (1998) | 18 dyslexics;  18 typical readers  Matched for age and intelligence | dyslexics M = 27.6,  18-41;  typical readers M = 24.5,  19-34 | Magnocellular visual temporal processing thresholds assessed using  2AFC 100% luminance contrast achromatic LED FFT task | Flicker Fusion thresholds were significantly lower in dyslexics (M = 52.8 Hz, SD = 0.87 Hz) as compared to controls (M = 57.1 Hz, SD = 1.18 Hz)  Combined performance on Flicker Fusion thresholds and motion coherence demonstrated 72.2% sensitivity and 83.3% specificity in discriminating dyslexic adults from controls. |
| (Edwards et al., 2004) | 21 dyslexics;  24 typical readers | dyslexics M = 11.17 (1.08);  typical readers M = 11.62 (1.49) | Magnocellular visual temporal processing thresholds assessed using a red LED chromatic flicker perception task, with separate experimental runs at high (100%) and low (10%) luminance contrast. Participants indicated when the light stopped or started flickering. | Low Contrast (10%) Flicker Fusion thresholds were not significantly lower in dyslexics (M = 24.44 Hz, SD = 5.91 Hz) as compared to controls (M = 23.57 Hz, SD = 2.97 Hz)  High Contrast (100%) Flicker Fusion thresholds were not significantly lower in dyslexics (M = 36.61 Hz, SD = 3.92 Hz) as compared to controls (M = 37.04 Hz, SD = 2.31 Hz) |
| McLean, Stuart, Coltheart, and Castles (2011) | 40 dyslexics;  42 typical readers | dyslexics  M = 9.5 (1.4);  typical readers  M = 9.6 (1.3) | Visual temporal processing thresholds assessed using a two-color red/green LED chromatic flicker perception task. Participants indicated when the light stopped or started flickering.  Parvocellular condition:  The frequency at which red/green color differentials were no longer discernable (i.e., ‘fused’ to a stable orange colour).  Magnocellular condition:  The magnocellular temporal threshold for isoluminant color flicker was recorded as the flicker frequency at which participants could no longer detect any flicker. | Parvocellular condition:  No differences between dyslexics (20.63 Hz) and controls (21.23 Hz)  Magnocellular condition:  Flicker Fusion thresholds were significantly lower in dyslexics (29.480 Hz) as compared to controls (30.838 Hz) |
| Brown, Peters, Parsons, Crewther, and Crewther (2020) | 18 dyslexics;  18 typical readers  Matched for age and intelligence | dyslexics  M = 10;04 (1;03);  typical readers  M = 10;06 (1;04) | Magnocellular visual temporal processing thresholds assessed using  two 4AFC achromatic LED FFT tasks at 75% and 5% luminance contrast | Low Contrast (5%) Flicker Fusion thresholds were significantly lower in dyslexics (M = 44.23 Hz, SD = 4.86 Hz) as compared to controls (M = 47.15 Hz, SD = 3.22 Hz)  High Contrast (75%) Flicker Fusion thresholds were significantly lower in dyslexics (M = 47.64 Hz, SD = 3.62 Hz) as compared to controls (M = 51.04 Hz, SD = 4.08 Hz) |

Supplementary Results:

Subgroup Characteristics: Identified Subgroups are not related to Double-Deficit Hypothesis Subtypes

The dyslexic sample were grouped into four subtypes according to the Double-Deficit Hypothesis to identify if subtypes predicted whether or not a dyslexic participant would have temporal processing impairments as based on the identified clusters. Participants were classified into the four subtypes based on whether they performed >1 SD below age-standardized norms on either rapid naming, phonological awareness, both tasks, or neither task. An ANOVA confirmed that the subtypes differed in accordance with the Double-Deficit Hypothesis (see Table S1). Results show that the Naming Speed Deficit and Double-Deficit subtypes had significantly poorer rapid naming performance than the Phonological and No Deficit Subtypes. As expected, the Naming Speed Deficit and Double-Deficit subtypes had comparable rapid naming performance. In comparison, the Phonological and Double-Deficit subtypes had significantly poorer phonological awareness performance than the Naming Speed Deficit and No Deficit Subtypes but performed comparably to each other. The groups also did not differ in age, nonverbal intelligence, reading rate or reading comprehension, but there were differences in reading accuracy, with the no-deficit subtypes performing significantly better than the other subtypes (See Supplementary Table S2).

To identify if the two clusters of dyslexics were associated with specific subtypes of DD, a logistic regression was performed. The presence (or absence) of a temporal processing deficit as based on the results of the cluster analysis was entered as the dependent variable, and the four subtypes (Phonological Deficit, Naming Speed Deficit, Double-Deficit, and No-Deficit) were entered as independent categorical predictors. The No Deficit Subtype was removed from the final model due to collinearity. The results of a direct logistic regression were not significant, χ^2^ (3, *N* = 54) = 4.88, *p* = .181, indicating that temporal processing impairments are not associated with specific subtypes. The model explained between 8.60% (Cox & Snell *R*^2^) and 11.50% (Nagelkerke *R*^2^) of the variance in temporal processing status, and correctly identified 64.80% of cases. As shown in Supplementary Table S3, none of the independent variables made a unique statistically significant contribution to the model.

| Supplementary Table S2.  *Univariate analyses comparing dyslexic subtypes on phonological and rapid naming performances, nonverbal intelligence, age, and reading performances* | | | | | | | | |
| --- | --- | --- | --- | --- | --- | --- | --- | --- |
|  | Subtypes | | | |  |  |  |  |
|  | Phonological Deficit (PD)  *n* = 10 | Naming Speed Deficit (NSD)  *n* = 12 | Double-Deficit (DD)  *n* = 18 | No-Deficit (ND)  *n* = 14 |  |  |  |  |
|  | *M (SD)* | *M (SD)* | *M (SD)* | *M (SD)* | *F* (3, 53) | *p* | *d* | Tukey HSD Post hoc |
| Rapid Naming | 93.44 (4.87) | 74.91 (8.54) | 76.06 (6.37) | 94.25 (5.83) | 31.60 | <.001 | 0.78 | NSD<PD**, NSD<ND**, NSD=DD, DD<PD**, DD<ND**, PD=ND |
| Phon. Awareness | 77.00 (5.87) | 94.54 (4.16) | 79.17 (3.93) | 101.23 (8.09) | 55.89 | <.001 | 0.67 | PD<NSD**, PD<ND**, PD=DD, NSD=ND, DD<NSD**, DD<ND** |
| Age | 10.71 (1.43) | 9.92 (1.10) | 10.35 (1.08) | 9.63 (1.13) | 2.02 | .122 | 0.69 |  |
| NVIQ | 101.30 (5.27) | 104.33 (8.94) | 106.17 (9.07) | 104.78 (7.48) | 0.78 | .507 | 0.43 |  |
| Reading Accuracy | 75.20 (5.73) | 76.75 (6.35) | 73.11 (5.19) | 84.31 (9.18) | 7.39 | <.001 | 1.34 | ND>PD*, ND>NSD*, ND>DD**, PD=NSD, PD=DD, NSD=DD |
| Reading Rate | 77.80 (10.79) | 73.91 (6.79) | 73.28 (6.64) | 80.15 (9.90) | 2.04 | .121 | 0.71 |  |
| Reading Comprehension | 92.30 (15.02) | 91.42 (22.72) | 90.17 (12.90) | 92.92 (11.31) | 0.08 | .967 | 0.14 |  |
| *Note*. **p* < .05, ** *p* < .001; Cohen’s *d* ≥ 0.2, *d* ≥ 0.5, and *d* ≥ 0.8, represent small, medium, and large effect sizes, respectively; all neuropsychological measures are reported as Standard Scores. | | | | | | | | |

| Supplementary Table S3.  *Logistic Regression Predicting Likelihood of a Temporal Processing Impairment from Double-Deficit Hypothesis Subtypes* | | | | | | | | |
| --- | --- | --- | --- | --- | --- | --- | --- | --- |
|  |  |  |  |  |  |  | 95.0% C.I for Odds Ratio | |
| Dyslexic Subtypes | B | S.E | Wald | *df* | *p* | Odds Ratio | Lower | Upper |
| Naming Speed Deficit | -0.92 | .81 | 1.31 | 1 | .253 | 0.39 | 0.08 | 1.94 |
| Phonological Deficit | -1.44 | .89 | 2.62 | 1 | .106 | 0.24 | 0.04 | 1.36 |
| Double-Deficit | 0.11 | .75 | 0.02 | 1 | .888 | 1.11 | 0.26 | 4.82 |
| No-Deficit | - | - | - | - | - | - | - | - |
| Constant | 0.59 | .56 | 1.11 | 1 | .292 | 1.80 |  |  |
| *Note:* The No Deficit Subtype was automatically removed from the regression model due to collinearity. | | | | | | | | |

| Table S4.  *Regression Equations and R^2^ values for Correlations between Flicker Fusion Thresholds (FFTs) and Reading Measures in Each Group (As Shown in Figure 2).* | | | |
| --- | --- | --- | --- |
|  | Phonological Awareness | Rapid Naming | Reading Rate |
| Low Contrast (5%) Flicker Fusion Thresholds | | | |
| MD-Dyslexics | Y = 0.08512*X + 34.46; *R*^2^ = 0.1064 | Y = 0.08393*X + 34.77; *R*^2^ = 0.09812 | Y = 0.1461*X + 31.43; *R*^2^ = 0.1173 |
| MT-Dyslexics | Y = 0.05420*X + 43.83; *R*^2^ = 0.03021 | Y = -0.1214*X + 58.28; *R*^2^ = 0.1095 | Y = -0.2285*X + 66.38; *R*^2^ = 0.2940 |
| Neurotypicals | Y = 0.05620*X + 41.40; *R*^2^ = 0.02972 | Y = 0.01783*X+ 45.50; *R*^2^ = 0.003633 | Y = 0.02502*X + 44.73; *R*^2^ = 0.004145 |
| High Contrast (75%) Flicker Fusion Thresholds | | | |
| MD-Dyslexics | Y = 0.1017*X + 36.54; *R*^2^ = 0.2597 | Y = 0.07337*X + 39.27; *R*^2^ = 0.1118 | Y = 0.08191*X + 39.01; *R*^2^ = 0.05548 |
| MT-Dyslexics | Y = 0.09221*X + 43.25; *R*^2^ = 0.07648 | Y = -0.1012*X + 60.12; *R*^2^ = 0.06831 | Y = 0.05778*X + 46.75; *R*^2^ = 0.01381 |
| Neurotypicals | Y = 0.1754*X + 32.29; *R*^2^ = 0.1838) | Y = 0.07383*X + 43.33; *R*^2^ = 0.03990 | Y = 0.1640*X + 34.26; *R*^2^ = 0.1218 |
